# Pathways to Student Motivation: A Meta-Analysis of Antecedents of Autonomous and Controlled Motivations

**DOI:** 10.3102/00346543211042426

**Published:** 2021-09-11

**Authors:** Julien S. Bureau, Joshua L. Howard, Jane X. Y. Chong, Frédéric Guay

**Affiliations:** Université Laval; Monash University; Curtin University; Université Laval

**Keywords:** academic motivation, psychological needs, autonomy support, self-determination theory, meta-analysis

## Abstract

Students’ self-determined motivation (acting out of interest, curiosity, and abiding values) is associated with higher academic well-being, persistence, and achievement. Self-determination theory posits that self-determined motivation is dependent on the satisfaction of three psychological needs (relatedness, competence, and autonomy), which are in turn facilitated through need-supportive behaviors from notable others. In this meta-analysis, conducted over 144 studies and more than 79,000 students, we sought to overview pathways to student motivation in order to verify (1) how do psychological needs rank in the strength of their prediction of self-determined motivation and (2) which autonomy-support providers (parents or teachers) are the most relevant for psychological need satisfaction in students and self-determined motivation. Results show that teacher autonomy support predicts students’ need satisfaction and self-determined motivation more strongly than parental autonomy support. In addition, competence is the most positive predictor of self-determined motivation, followed by autonomy and then by relatedness.

Students’ motivation is relevant for the quality of their learning experience (Pintrich & De Groot, 2003). Self-determination theory (SDT) is a comprehensive motivational framework that has been used to explain how individuals thrive within many life domains (Ryan & Deci, 2017). Specifically, SDT has proven effective at qualitatively and quantitatively describing types of motivation and their potential impact on achievement and other desirable school outcomes (Howard et al., 2021; Vasconcellos et al., 2020). The degree to which students experience these types of motivation is expected to vary according to situational and environmental factors (Vallerand, 1997), making motivation a primary target for intervention. According to SDT, students will experience self-determined motivation when behaviors from people they interact with satisfy their psychological needs of autonomy, competence, and relatedness (Ryan & Deci, 2017). However, the theory makes no proposition about whether each need contributes equally or whether some will be more relevant than others.

In this meta-analysis, we sought to verify (1) which psychological need is the strongest predictor of self-determined motivation and (2) which autonomy-support provider (parents, teacher) is the most relevant for psychological need satisfaction and self-determined motivation. Consequently, this study seeks to test core principles in SDT to advance our understanding of the different pathways in which teachers and parents may affect students’ psychological need satisfaction and self-determined motivation.

## Student Motivation According to Self-Determination Theory

Student motivation is defined within SDT along a continuum of motivation quality that brings varying levels of self-determination (i.e., being at the origin of one’s action; Ryan & Deci, 2017). In decreasing levels of self-determination, there is intrinsic motivation followed by three types of extrinsic motivation, namely, identified, introjected, and external regulations. When acting out of intrinsic motivation, the reasons for engaging in learning are tied to inherent enjoyment and interest in doing academic tasks. This represents the highest level of self-determination. In contrast, with extrinsic motivation, the reasons for engaging in learning are tangential to learning such that it is rather instrumental for achieving other benefits. Identified regulation represents a state that drives students to engage in school activities that they consider personally important and meaningful. It implies a high level of self-determination, albeit not as high as when intrinsically motivated. Identified and intrinsic motives together represent autonomous motivation. Introjected regulation, in turn, depicts a state that drives students to participate in their schooling to avoid feelings of shame and guilt or to assert pride. This regulation implies a moderate level of self-determination. External regulation represents a state where students’ involvement in school activities is driven by factors outside individuals’ control, such as external rewards or sanctions to avoid. External regulation conveys low levels of self-determination. External and introjected regulations together represent controlled motivation. Last, a final form of (non-)motivation is sometimes assessed within academic motivation scales, namely, amotivation. It represents a state where students see no clear reason to pursue school activities, and it is considered a non-self-determined form of motivation (Vallerand et al., 1992). Integrated regulation is an additional motivation type representing a state of significant congruence between school engagement and one’s values and needs. While it is theoretically situated between intrinsic and identified motives in terms of self-determination, researchers agree that this form of regulation requires individuals to have formed a coherent identity (Deci et al., 1996) before developing. Consequently, this type of extrinsic motivation is rarely assessed in studies on children and adolescents. Consequently, very few student motivation scales include it.

Recent meta-analyses have demonstrated that motivation types related differently to a wide range of outcomes across education and physical education fields of research (Howard et al., 2020; Howard et al., 2021; Toste et al., 2020; Vasconcellos et al., 2020). For example, intrinsic and identified motives (i.e., autonomous motivation) were found to be strong positive predictors of students’ achievement, engagement, positive affect, goals, and self-esteem. Introjected regulation demonstrated mixed effects, relating positively to desirable and undesirable outcomes, while external regulation demonstrated primarily nonsignificant results or positive associations with maladaptive outcomes. Amotivation related positively to undesirable outcomes. Given the consistent relations between the various motivation types and student outcomes, and acknowledgement that student motivation can benefit from outside interventions (Lazowski & Hulleman, 2016), it becomes important to identify factors with the potential to foster or hinder the various types of motivation.

## Psychological Needs According to Self-Determination Theory

Self-determination theory details three fundamental psychological needs that are purported to be essential for individuals’ daily functioning, including a role in cultivating self-determined motivation types. The specified needs are those of autonomy, competence, and relatedness (Ryan & Deci, 2000). Students experiencing autonomy perceive that they are engaging in learning tasks freely and voluntarily, without perceived coercion. Students experiencing competence are confident that their actions are impactful in shaping their academic experience. Students experiencing relatedness feel connected with important others in their school (e.g., teachers, friends; Baumeister & Leary, 1995; Chen et al., 2015). The three psychological needs are best satisfied when students benefit from need-supportive contexts. SDT asserts the importance of supportive interpersonal relationship (e.g., with teachers and parents) for fostering students’ need satisfaction (Chirkov & Ryan, 2001; Ryan & Deci, 2020).

Although need support can be directed toward the satisfaction of either autonomy, competence, or relatedness needs, the bulk of research on need support has focused on autonomy support as it has been found to be important for fostering not only autonomy need satisfaction but also competence and relatedness (Sheldon et al., 2009). Autonomy-supportive behaviors promote students’ volitional functioning (Carpentier & Mageau, 2019; Reeve & Jang, 2006): these are empathy, providing information, and supporting active participation. Teachers and parents acting with empathy take the student’s/child’s perspective and acknowledge their individuality and feelings. Teachers and parents who provide information give explanatory rationales for rules and demands, supporting internalization of the importance of schoolwork. Teachers and parents who support an active participation provide meaningful self-relevant decision-making opportunities, cultivating intrinsic motivation (Mageau et al., 2015; Reeve & Cheon, 2021). These behaviors encourage self-determination in students/children, and make them feel competent and connected to the teacher/parent, catering to all three psychological needs. Meta-analyses on autonomy-supportive teaching interventions (Reeve & Cheon, 2021) and on correlates of autonomy-supportive teaching in the physical education context (Vasconcellos et al., 2020) point toward the numerous benefits of this teaching style for students in terms of affective, cognitive, and behavioral outcomes, in addition to their positive effects on need satisfaction. For example, autonomy-supportive teaching increases classroom engagement (Cheon et al., 2019), initiative (Reeve et al., 2020), and achievement (Cheon et al., 2020), as well as motivation for out-of-school activities (Hagger & Chatzisarantis, 2016). A meta-analysis on parental autonomy support and its relations to educational and psychosocial outcomes in children (Vasquez et al., 2016) has associated it with positive educational outcomes such as students’ achievement, perceived competence, positive attitudes toward school, and self-regulation. Other autonomy-supportive sources such as peers or school administration have also been examined in past research (Hang et al., 2015; Zhou et al., 2019), showing that they generally have an incremental positive effect on motivation and adjustment, but they have received less scholarly attention.

Taken together, previous research has demonstrated the extended benefits of teacher and parental autonomy support for students. A central premise of SDT asserts a causal sequence that links autonomy support with enhanced psychological need satisfaction, which in turn fosters high-quality motivation, which in turn fosters adaptive outcomes. While the motivation-to-outcomes part of this sequence was recently meta-analyzed in the field of education (Howard et al., 2021), a systematic and simultaneous test of the first part of the sequence from autonomy-support to psychological needs, and then from needs to motivation, has never been conducted.

## Prediction of Student Motivation by Psychological Needs and Autonomy Support

Previous research shows consistent support for need satisfaction at school being a paramount factor in predicting and understanding self-determined student motivation (Deci et al., 1996). As students experience psychological need satisfaction, they begin an internalization process where reasons for engaging in school tasks shift from less self-determined (e.g., because it is mandatory) to more self-determined (e.g., because they enjoy it; Deci et al., 1994; Ryan & Deci, 2017). This internalization process explains why experiencing need satisfaction in a setting will ultimately translate into valuing the activity more (identified regulation) and showing interest for it (intrinsic motivation). Results from a previous meta-analysis in physical education (Vasconcellos et al., 2020) showed that all needs related to motivation types in highly similar ways: they had strong positive relations with intrinsic motivation and identified regulation, moderate positive relations with introjected regulation, weak negative relations with external regulation, and moderate negative relations with amotivation. Using meta-analytic path analysis to uncover unique variance predicted by individual needs, this study showed that competence was the strongest predictor of self-determined motivation followed by autonomy, whereas relatedness had only a small contribution. Although this study implies that competence plays a somewhat stronger role in fostering self-determined motivation for physical education, this is not true for all domains. A recent meta-analysis of need satisfaction outcomes in the workplace demonstrated that autonomy was the most influential of the three needs in predicting work motivation, explaining as much as 42% of the total variance in intrinsic motivation (Van den Broeck et al., 2016). It is therefore important to explore the education literature more broadly and investigate how psychological needs will differentially predict motivation types for students.

In addition to basic psychological needs, autonomy support from teachers has also been positively associated with self-determined types of motivation (intrinsic, identified) and negatively associated to motivation types closer to the non-self-determined pole (introjected, external, amotivation; Reeve & Cheon, 2021). With regard to parental autonomy support, it has been linked positively to autonomous motivation, but to more controlled forms of motivation as well (Guay et al., 2013; Vasquez et al., 2016). In general, autonomy-supportive environments created by teachers and parents bring students to value and enjoy schoolwork, fostering self-determined school motivation types (Aelterman et al., 2019; Chirkov & Ryan, 2001). However, SDT does not specify whether one provider of autonomy support should be more important than the other in predicting motivation, or if their pathways in doing so vary. Finally, while SDT and the hierarchical model of motivation (Vallerand & Ratelle, 2002) predict a sequence from autonomy support to needs, and then from needs to motivation, there is no specific indication regarding the relative strength of these mediated effects.

## The Present Study

The main objective of this research was to meta-analytically test the motivational sequence theorized by SDT linking autonomy support, need satisfaction, and types of student motivation. In doing so, we want to achieve a better understanding of the associations proposed by SDT and ultimately lead to a refined understanding on how to foster adaptive educational outcomes. To do so, we gathered and documented all available studies set within the educational psychology literature linking academic motivation and its antecedents as specified by SDT, namely, need satisfaction and autonomy support. We first calculated meta-analytically derived correlations for all associations between motivation types, needs, and autonomy support variables. Second, we used relative weight analysis to evaluate the unique predictive strength of individual needs. Third, we applied meta-analytic structural equation modeling to test a mediation model from autonomy support to need satisfaction, and then from needs to motivation types, allowing for comparing the predictive strength of both autonomy support types, as well as testing the relative strength of the mediation effects. Last, to investigate how the studied associations were shaped by individual characteristics, the moderating influences of age and gender are investigated. Both have explained variations in how motivation types predict adaptive and maladaptive outcomes in previous research (Howard et al., 2021; Vasconcellos et al., 2020) implying that associations between academic motivation and educational variables is gender- and age-dependent. Education context (classroom or physical education), scale used, and publication status were also included as moderators to understand if any contextual or methodological bias could increase or decrease the strength of the relations tested.

## Method

### Inclusion Criteria

We employed three inclusion criteria while collecting data: (1) data must be based on students ranging from primary school to university (inclusive); (2) authors must have reported at least one correlation between a motivation type and a need satisfaction or autonomy support variable^1^; (3) data must be based on validated scales measuring motivation types, meaning that the scale used had to refer to a validation paper or present reliability statistics as well as evaluate different types of extrinsic motivation. Three validated scale “families” were identified: the Self-Regulation Questionnaire (SRQ, PLOC, ASRQ; Ryan & Connell, 1989), the Academic Motivation Scale (AMS; Vallerand et al., 1992), and the Behavioral Regulation Questionnaire (BREQ, BREQ-2, BREQ-2r, BRSQ; Mullan et al., 1997). In addition, two other relevant validated scales were found: the Situational Motivation Scale (SIMS; Guay et al., 2000) and the Exercise Motivation Scale (EMS; Li, 1999). These have been used in numerous studies and are validated across multiple cultural contexts, age groups, and grade levels. Articles that used their own adapted measure of motivation were included if they met the previously mentioned criteria.

### Literature Search

**Figure 1.**
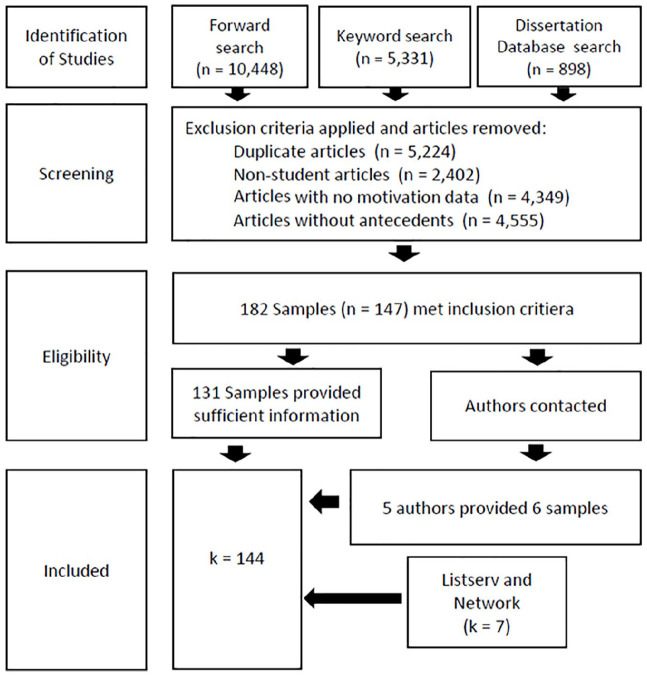
Flow diagram representing literature search procedures and application of exclusion criteria. *Note*. *n* denotes the number of articles; *k* denotes number of samples.

In searching the literature, we applied four primary methods to obtain all relevant published and unpublished data. Gray literature was included at all stages, including dissertation, conference presentations, and other unpublished work. The first, second, and third authors first conducted forward searches collecting all publications which had cited the scale validation studies for each of the included scales through Google Scholar. Second, the EBSCO and PsycINFO databases were searched by the second author, using the search terms “self-determination” and “student” as well as by searching for the specific scales (see aforementioned inclusion criteria). Third, the second author used the same search terms to search the Proquest Dissertation and Thesis Global database. Finally, the first and second authors advertised for unpublished data through several mailing lists (i.e., those administrated by the Center for Self-Determination Theory, the American Educational Research Association, the Society of Personality and Social Psychology, and the Society for the Study of Motivation). Each potential sample identified through these procedures was examined by the second and third authors, as well as by a research assistant. When studies did not contain all relevant effect size information, corresponding authors were contacted directly by the second author. Studies not meeting the inclusion criteria were excluded, and duplicate samples were removed at this stage. Figure 1 depicts this process. The complete list of references used in the meta-analysis is presented in the online supplemental materials.

The final database consisted of 144 samples (98 published, 47 unpublished), including a total of 1,718 correlation coefficients from 79,079 participants (each sample ranging from 26 to 4,566 participants, mean = 543). The mean age of students across samples was 16.55 years old, and the average proportion of males in each sample was 45.46%. Sample characteristics are presented in Table S1 in the online supplement.

### Coding

A coding spreadsheet was developed by the second author including all information concerning motivation variables and covariates (i.e., type of motivation or covariate, scale used to collect data, and reliability of measures). Correlations between motivation types and predictors were coded as the primary effect size data. Sample size and demographic information pertaining to country and language in which data were collected, participant age, and percentage of males in the sample were also recorded. Publication status was also coded where theses, dissertations, and conference presentations were coded as unpublished. In order to assess and ensure the reliability of coding, the second and third authors double-coded approximately 10% of data. Interrater agreement was very high (Cohen’s κ = .95; McHugh, 2012), and disagreements were discussed by the second and third authors. Data were examined for coding errors by the first and second authors prior to analysis.

### Meta-Analytic Procedures

Meta-analytic calculations were conducted using the “robumeta” package within the R software (Fisher & Tipton, 2015), and random-effects models were applied throughout (Hunter & Schmidt, 2000). Corrections for reliability were performed prior to any analysis. When alpha coefficients were missing, mean coefficients were imputed. Non-independent data were adjusted through robust variance estimator (Hedges et al., 2010). This method adjusts scores in non-independent samples to avoid sample size inflation. Cumulative analysis and one-study-removed analyses were conducted in order to examine potential outliers (Borenstein et al., 2009). As no study was found to substantially change any parameter, all studies were kept in the analyses. At this stage, predictor variables with too few samples (<5) to analyze reliably were removed from the study. This included the variables of peer autonomy support, relationship quality (between student and teacher, peers, and parents), and need frustration variables (autonomy, competence, and relatedness frustration).

Meta-analytic correlations (ρ) are reported along with the associated 95% confidence intervals, number of samples included in analysis (*k*), standard error of the estimate, and heterogeneity statistics. Specifically, *T*^2^ and *I*^2^ statistics are provided to examine heterogeneity in which *T*^2^ estimates the variance of effect-size parameters and *I*^2^ estimates the proportion of this variance that is likely to be attributable to true moderating influences and not due to sampling error, statistical artifacts, or chance (Higgins et al., 2003). An *I*^2^ score greater than 75% indicates a high likelihood of unaccounted moderating factors, whereas a score lower than 25% indicates a relatively low likelihood of meaningful moderating factors (Higgins et al., 2003).

Relative weight analysis (RWA) was conducted with the R software package (Tonidandel & LeBreton, 2015). Using meta-analytic associations between needs and motivation types, this analysis estimates how much variance in outcomes could be uniquely attributed to each predictor if multicollinearity was suppressed. Specifically, RWA involves the creation of a new set of orthogonal predictors as similar as possible to the original set of predictors. The outcome variable is then regressed onto these new orthogonal predictors, and that regression is weighted according to the similarity between the original and orthogonal predictors, yielding an estimate of the unique variance explained by predictors.

**Figure 2.**
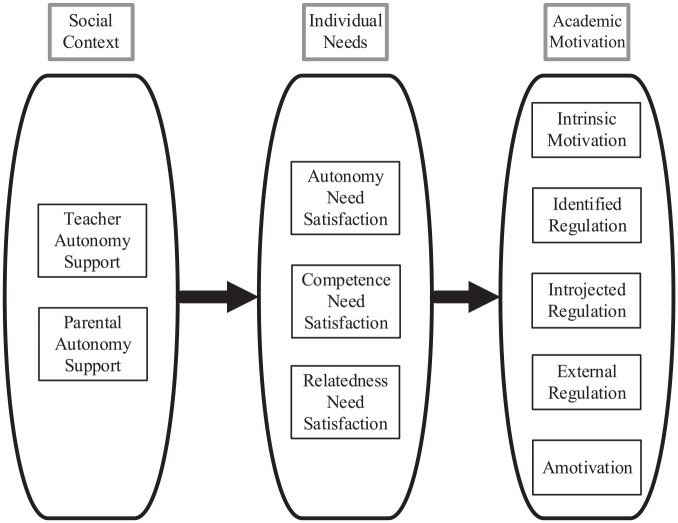
Hypothesized path model.

We further tested our hypothesized model (see Figure 2), through meta-analytic path analysis following a weighted-covariance generalized least squares procedure with multiple imputation (W-COV GLS with MI; Lv & Maeda, 2020). This multivariate procedure was preferred over univariate MASEM (meta-analytic structural equation modeling) because of its capacity to consider the dependency among same-study correlations when pooling the covariance matrix for the MASEM (Lv & Maeda, 2020). In contrast, univariate MASEM or meta-analytic path analysis will consist of generating a pooled correlation matrix from meta-analytic correlations directly, yielding biased estimates and higher chances of having a nonpositive definite correlation matrix (Cheung, 2015). The W-COV GLS method was also preferred over other techniques (TSSEM, OSMASEM; Cheung & Chan, 2009; Jak & Cheung, 2020) because of its ability to fit a structural model when there are multiple missing correlations (73.3% missing correlations in the present case; Jak & Cheung, 2018; Lv & Maeda, 2020). The W-COV GLS procedure with MI consists of gathering all correlation matrices from the included studies and using averaged multiple imputation to replace the missing correlations in each matrix.^2^ Then, a pooled correlation matrix is computed on which the structural model is tested. In this study, the structural model was fully identified, which yields no fit statistic.

Finally, several potential moderating variables were examined. Moderating influences of both the educational context of the study (i.e., classroom education or physical education) and scale used (AMS, PLOC, BREQ) were examined through subgroup analyses. Age and gender were investigated through meta-regression. Meta-regression indicates how much of the unexplained heterogeneity (*I*^2^) can be accounted for by the potential moderating variable. The regression coefficient associated with this analysis indicates the estimated change in effect size for a one-point increase in the moderator. A *R*^2^ statistic as well as the statistical significance estimate associated with the regression coefficient are also provided. A last potential moderator, publication bias, was examined through multiple approaches. Trim and Fill procedures were applied to examine the symmetry of observed effects and determine if studies were likely to be missing due to publication bias (Duval & Tweedie, 2000). This analysis indicates the number of studies estimated to be missing and provides a corrected estimate of the effect size, thereby indicating the likelihood of publication bias (Duval & Tweedie, 2000). Egger’s test of the intercept was also applied (Egger et al., 1997). This test also examines the symmetry of observed effects and, through regression, determines if there is significant publication bias. Last, subgroup analyses were also conducted to directly compare the estimated effect sizes from published and unpublished data. In these analyses, similar estimated effects and overlapping confidence interval would indicate that published and unpublished samples are similar (Cumming & Finch, 2005) and therefore do not present evidence for publication bias.

## Results

Correlation analyses (see Tables 1 and 2) indicated that each psychological need generally showed stronger correlations with more self-determined types of motivation. Specifically, autonomy related negatively with amotivation (ρ = −.34, *k* = 29), was unrelated to external regulation (ρ = −.04, *k* = 46), and correlated positively with introjected (ρ = .23, *k* = 42), identified (ρ = .48, *k* = 42), and intrinsic motivation types (ρ = .57, *k* = 52). Furthermore, correlations between autonomy and motivation types were all significantly different from each other as indicated by non-overlapping confidence intervals (Cumming & Finch, 2005). A similar pattern arose for competence with a negative association to amotivation (ρ = −.38, *k* = 37), no relation to external regulation (ρ = −.01, *k* = 57), and positive correlations to introjected, identified, and intrinsic motivation types (ρ = .23, .53, and .58, respectively, *k* = 58, 57, 69). Confidence intervals of associations between competence and the various motivation types did not overlap, indicating significant differences throughout. The need for relatedness was also found to demonstrate similar patterns (in the same ordering: ρ = −.38, −.02, .23, .53, and .58, respectively, *k* = 29, 45, 45, 44, 56), and only associations between relatedness and identified and intrinsic motivation types had overlapping confidence intervals.

**Table 1:** Meta-analytic correlations between basic psychological needs, autonomy support and motivation types

Covariate	*k*	*N*	ρ	95% Confidence interval		*SE*	*T* ^2^	*I* ^2^
Motivation				Lower	Higher			
Autonomy								
Amotivation	29	13,439	−.34	−.44	−.23	.05	.10	97.81
External	46	18,842	−.04	−.12	.04	.04	.09	97.17
Introjected	42	17,920	.23	.17	.29	.03	.04	94.86
Identified	42	18,395	.48	.41	.56	.04	.04	94.50
Intrinsic	52	23,759	.57	.50	.63	.03	.04	94.88
Competence								
Amotivation	37	20,579	−.38	−.47	−.30	.04	.06	97.07
External	57	34,558	−.01	−.08	.05	.03	.06	97.02
Introjected	58	34,781	.23	.19	.28	.02	.02	93.49
Identified	57	34,705	.53	.47	.58	.03	.03	94.61
Intrinsic	69	40,789	.58	.53	.63	.02	.03	94.05
Relatedness								
Amotivation	29	13,885	−.30	−.38	−.23	.04	.04	95.04
External	45	20,175	.01	−.05	.07	.03	.06	96.29
Introjected	45	20,175	.21	.15	.26	.03	.04	94.37
Identified	44	20,338	.44	.39	.49	.02	.02	91.73
Intrinsic	56	27,209	.44	.39	.48	.02	.03	93.15
Teacher Autonomy Support								
Amotivation	19	8,640	−.32	−.38	−.26	.03	.02	86.90
External	34	21,792	−.10	−.17	−.02	.04	.03	95.15
Introjected	35	22,103	.17	.13	.21	.02	.01	87.82
Identified	44	28,515	.44	.39	.49	.02	.03	95.22
Intrinsic	47	33,517	.48	.43	.54	.03	.05	96.91
Parent Autonomy Support								
Amotivation	6	4,810	−.23	−.34	−.12	.04	.01	83.25
External	15	8,859	.05	−.03	.13	.04	.01	88.81
Introjected	15	8,859	.15	.06	.24	.04	.02	92.49
Identified	15	8,859	.28	.22	.34	.03	.01	83.52
Intrinsic	12	5,549	.23	.15	.31	.04	.01	79.32

*Note*. *k* = number of samples; ρ = correlation after correction for reliability and weighted by samples size. As some studies reported autonomy support from both mothers and fathers, the number of correlations entered into RVE analyses was greater than the number of samples; amotivation (8), external (19), introjected (19), identified (19), and intrinsic (16). However, the number of independent effect analyzed is congruent with *k*.

**Table 2:** Correlations between basic psychological needs

	Autonomy	Competence	Relatedness
Autonomy	—	58	55
Competence	.768	—	56
Relatedness	.648	.638	—

*Note*. Meta-analytic correlations below the diagonal. Number of included samples above the diagonal.

Autonomy support from teachers followed the same general pattern relating more positively with self-determined types of motivation. Specifically, whereas teacher autonomy support was significantly and negatively related to amotivation (ρ = −.32, *k* = 19) and external regulation (ρ = −.10, *k* = 34), it had positive associations to introjected (ρ = .17, *k* = 35), identified (ρ = .44, *k* = 44), and intrinsic motivation types (ρ = .48, *k* = 47). Autonomy support from parents displayed similar results with a negative correlation with amotivation (ρ = −.23, *k* = 6), but no relation to external regulation (ρ = .05, *k* = 15). Relations connecting parental autonomy support to introjected (ρ = .15, *k* = 15), identified (ρ = .28, *k* = 15), and intrinsic motivation (ρ = .23, *k* = 12) were all positive and significant. Although the point estimates of the correlations differ, only the association between parental autonomy support and amotivation was different from the associations with all other motivation types. The association with external regulation was also different from those with identified and intrinsic motivation types.

Relative weight analysis was used to estimate how psychological needs predicted unique variance in motivation types once the shared variance among needs was accounted for (Table 3). With regard to amotivation (*R*^2^ = .15; all negative associations), about half of explained variance was weighted on competence (47%), while autonomy (30%) and relatedness (23%) shared the remaining variance. For external regulation, variance explained by needs was negligible (*R*^2^ = .004), making the relative weight analysis meaningless. The three needs equally predicted introjected regulation (35%, 36%, and 29%, respectively, for autonomy, competence, and relatedness; *R*^2^ = .06; all positive associations). Identified regulation (*R*^2^ = .30) was most strongly predicted by competence (44%), followed by autonomy (30%) and relatedness (26%). Finally, intrinsic motivation (*R*^2^ = .38) was predicted in a similar way by autonomy (39%) and competence (42%), but relatedness was less important (18%).

**Table 3:** Relative weight (RW) analysis of need satisfaction predicting motivation

Outcome	*R* ^2^	Autonomy, RW (%)	Competence, RW (%)	Relatedness, RW (%)
Amotivation	.15	30.34	46.90	22.77
External	.004	62.43	14.27	23.30
Introjected	.06	35.16	35.58	29.27
Identified	.30	29.96	44.38	25.66
Intrinsic	.38	39.30	42.33	18.38
Average (weighted by R2)		34.49	43.21	22.31

**Figure 3.**
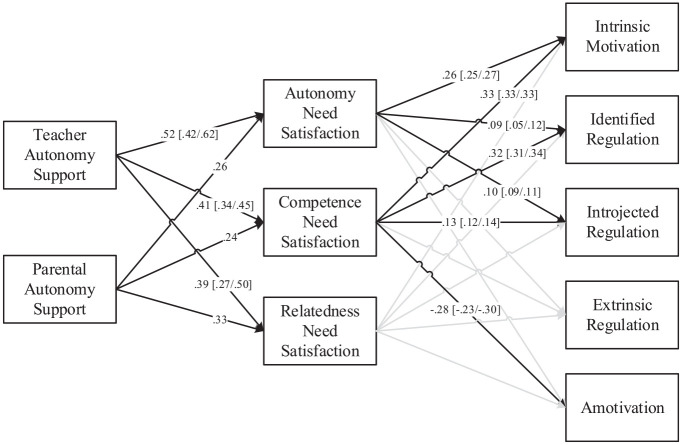
Obtained path model. *Note*. All paths shown are averages between the three models, with minimum and maximum values shown. Direct paths from need support to motivation variables were left out for clarity purposes. Paths below .10 in all models are shown in lighter gray.

We computed a weighted-covariance generalized least squares meta-analytic structural equation modeling with multiple imputation (W-COV GLS MASEM with MI; Lv & Maeda, 2020). This analysis tested this model: autonomy support from teachers and parents → need satisfaction → academic motivation types. Results are presented in Figure 3. The direct links from autonomy-supportive behaviors to motivation types were also tested. Unfortunately, the number of samples that included parental autonomy support were insufficient (*M_k_* = 8.89) to compute imputed values for these correlations (van Buuren & Groothuis-Oudshoorn, 2011). Since it was not possible to run a model with all 10 variables using MI, three models were computed. A first model (Model 1; *k*_min_ = 2, *k*_max_ = 71, *M_k_* = 38.4, *SD_k_* = 21.5) included all 10 variables, used the same W-COV GLS MASEM approach, but used pairwise deletion (PD; Lv & Maeda, 2020) instead of multiple imputation. A second model (Model 2; *k*_min_ = 7, *k*_max_ = 71, *M_k_* = 45.8, *SD_k_* = 17.1) used the W-COV GLS MASEM with MI approach but did not include parental autonomy support. For comparison purposes, a third model (Model 3; *k*_min_ = 7, *k*_max_ = 71, *M_k_* = 45.8, *SD_k_* = 17.1) used the W-COV GLS MASEM with PD, as in Model 1, and did not include parental autonomy support, as in Model 2. Model results are presented in Table 4.

**Table 4:** Meta-analytic structural equation modeling results

Model	Outcome	*R* ^2^	Antecedent				
			Teacher AS	Parent AS	Autonomy	Competence	Relatedness
1 PD—All variables	Autonomy	.33	.42	.26			
	Competence	.24	.34	.24			
	Relatedness	.26	.27	.33			
	Amotivation	.14	−.13 (−.10)	.05 (−.08)	.06	−.30	−.08
	External	.01	−.05 (−.03)	.08 (−.01)	−.05	−.07	.05
	Introjected	.07	.03 (.08)	.07 (.05)	.09	.13	−.01_n.s._
	Identified	.32	.19 (.17)	−.01_n.s._ (.13)	.12	.31	.08
	Intrinsic	.36	.07 (.24)	−.06 (.16)	.27	.33	.05
2 MI—No parent AS	Autonomy	.38	.62	—			
	Competence	.20	.45	—			
	Relatedness	.25	.50	—			
	Amotivation	.16	−.18 (−.14)	—	.01	−.23	−.08
	External	.01	−.08 (.02)	—	.01_n.s._	−.05	.07
	Introjected	.07	.04 (.14)	—	.11	.12	.05
	Identified	.33	.19 (.24)	—	.05	.34	.11
	Intrinsic	.40	.13 (.32)	—	.25	.33	.03
3 PD—No parent AS	Autonomy	.28	.53	—			
	Competence	.20	.44	—			
	Relatedness	.16	.41	—			
	Amotivation	.13	−.12 (−.12)	—	.07	−.30	−.07
	External	.01	−.03 (−.02)	—	−.04	−.07	.07
	Introjected	.06	.05 (.12)	—	.10	.14	.01_n.s._
	Identified	.32	.18 (.23)	—	.11	.31	.07
	Intrinsic	.36	.06 (.30)	—	.26	.33	.03

*Note*. All models use a weighted covariance generalized least squares pooling method for the correlation matrix and a ML estimator for fitting the SEM model. Models are fully identified (no fit indices provided). *N* for Models 1 and 3 are harmonic mean (9,079 and 13,812, respectively), *N* for Model 2 is the sum of total participants (76,477; Lv & Maeda, 2020). Coefficients are direct effects. Indirect effects through all needs globally are in parentheses. Unless otherwise stated, all coefficients are significant at the *p* < .01 level. AS = autonomy support; PD = pairwise deletion; MI = multiple imputation.

Across the three models, intrinsic motivation was the motivation type with the most explained variance (mean *R*^2^ = .37), followed by identified regulation (mean *R*^2^ = .32) and amotivation (mean *R*^2^ = .14). Mean *R*^2^ for introjected (.07) and external regulation (.01) were much smaller. Competence was the strongest predictor of amotivation (β = −.23 to −.30), identified regulation (β = .31 to .34), and intrinsic motivation (β = .33). Autonomy also predicted intrinsic motivation (β = .25 to .27). Teacher autonomy support, in turn, was a moderately strong predictor of autonomy (β = .42 to .62), competence (β = .34 to .45), and relatedness (β = .27 to .50). In terms of predicting motivation types, teacher autonomy support predicted, with both direct and indirect effects through the three needs, amotivation (β = −.12 to −.18; β_indirect_ = −.10 to .14), identified regulation (β = .18 to .19; β_indirect_ = .17 to .24) and intrinsic motivation (β = .07 to .13; β_indirect_ = .24 to .32). Prediction of introjected and external regulation from teacher autonomy support were weaker, although there were consistent small positive indirect effects on introjected regulation (β_indirect_ = .08 to .14). In the model including both teachers and parental autonomy support, direct and indirect paths from teacher autonomy support to self-determined motivation types and amotivation were stronger than those from parental autonomy support. In fact, all direct predictions from parental autonomy support to motivation types were close to null when teacher autonomy support and needs were taken into account. Parental autonomy support only accounted for some indirect prediction in identified and intrinsic motivation types (β_indirect_ = .13 and .16, respectively).

Moderation analyses were conducted on meta-analytic correlations to examine the role of student age, gender, educational context, and scale used on the results, as well as the possibility of publication bias (see Tables S2 to S5 in the supplemental materials for detailed results). Two moderating effects were noticed based on student age. First, it appeared that associations between autonomy support from parents and amotivation, identified regulation and intrinsic motivation diminished as students grew older. A second pattern concerns the prediction of amotivation by the needs for autonomy and competence. For these two relationships, the correlation became more negative as mean age in the sample increased, indicating that needs became more predictive of amotivation as students’ age increased. Only one notable effect was moderated by gender. When the proportion of males in a sample increased, the correlation between competence and both identified and intrinsic motivation types increased. This indicates that competence may relate to identified and intrinsic motives more strongly for males than for females.

Regarding education context (i.e., classroom education vs. physical education), the correlations between amotivation and both autonomy and relatedness is substantially more negative in the classroom samples as compared to the physical education samples. However, the relationship between competence and amotivation is consistent across these groups. It is also worth noting that the associations between predictors and identified and intrinsic motives were generally higher in PE classes. When comparing results from various motivation scales (the AMS, the PLOC, and the BREQ), identified and intrinsic motivation types measured through the AMS had generally weaker positive relations with psychological needs and autonomy support than when measured with the PLOC or BREQ. Furthermore, the strength of the relations between external regulation and various antecedents were different across scales. While external regulation measured with the AMS shows weak positive associations with needs and autonomy support, the other scales rather show negative associations between needs or autonomy support and external regulation.

Finally, tests of publication bias were conducted which showed some evidence of bias. Specifically, several relationships involving teacher autonomy support may be influenced by publication bias. For this variable, trim and fill analysis as well as Eggar’s regression test both indicated potential missing studies that could have found smaller or nonsignificant relationships between teacher autonomy support and three types of motivation (introjected, identified, and intrinsic). It was estimated that their true effect sizes be potentially closer to .09, .29, and .29, as opposed to results from the collected data which estimate these effects at .17, .44, and .48. Subgroup analysis found similar differences with published studies reporting on average higher effects sizes than unpublished studies in regard to these three correlations, though differences were not as large in this analysis. Although this potential case of publication bias suggests smaller effect sizes, it should be noted that effects would remain in the same direction and lead to similar interpretation.

## Discussion

Self-determination theory specifies that need-supportive contexts should lead to highly self-determined student motivation. Autonomy supportive behaviors from teachers and parents are thus expected to foster self-determined motivation through the satisfaction of students’ psychological needs. This sequence has been supported by a large body of research that has been synthesized and meta-analyzed in this research. The current research aimed to comprehensively test the need-based pathways through which teachers and parents are able to influence student motivation in order to identify intervention targets through which various types of motivation could be facilitated and sustained. It complemented previous meta-analytic investigations on student motivation that were either restricted to physical education (Vasconcellos et al., 2020) or focused on outcomes of student motivation (Howard et al., 2021). Results globally show that autonomy-supportive behaviors from teachers and parents and individual needs relate in similar ways to motivation types. However, their unique predictive strength also varies greatly, leading to interesting implications.

### The Roles of Competence and Autonomy

With regard to needs and motivation, competence seems to be the driving factor in predicting intrinsic motivation, identified regulation, and amotivation. A previous meta-analysis (Howard et al., 2021) has shown that intrinsic motivation, identified regulation, and amotivation were the motivation types with the strongest predictive power in their associations with student achievement, engagement, well-being, and self-evaluation outcomes. The current results thus concur with recent studies positioning the need for competence as the central and most important need for students (Levesque-Bristol et al., 2020). Furthermore, these results concur with Vasconcellos et al.’s (2020) findings that competence need satisfaction has the strongest association with students’ autonomous motivation and amotivation in the physical education context. The current results drawn from both RWA and MASEM showed the need for autonomy to be mostly effective in predicting intrinsic motivation, to a similar degree as the need for competence, but much less in predicting other motivation types. This is notable as a recent meta-analysis in the work context (Van den Broeck et al., 2016) showed that the need for autonomy was the driving factor in explaining motivation types and work outcomes. This does not seem to be the case in education as both our results and those of Vasconcellos et al. (2020) show autonomy to be secondary to competence in predicting academic motivation. This difference between the two contexts may be due to the saliency of achievement in the education context. Despite work output also being subject to evaluations by organizations, those appraisals remain secondary to working tasks. In contrast, evaluations in school are central in almost every aspect of students’ experience, serving the multiple purposes of motivating schoolwork, determining how much learning occurred, ranking students, and establishing their potential for future academic and professional endeavors. Those context-differing results could also be due to a greater personal weight awarded to the need for autonomy as we age, a hypothesis that has been advanced in both the educational (Ratelle et al., 2007) and work contexts (Truxillo et al., 2012). However, such a moderation effect was not found in our results.

### The Role of Relatedness

When compared with students’ autonomy and competence, students’ relatedness was a weaker factor in its association with all motivation types. Even though relatedness remains a positive factor in explaining types of self-determined motivation, it seems that feelings of belongingness and meaningful connections at school may not be as important for student motivation as it could be for other facets of the student experience (e.g., intention to leave the program; Hilts et al., 2018). This finding also aligns with meta-analyses from other contexts which consistently find relatedness to be a positive antecedent of motivation types with smaller effect sizes compared with needs for autonomy and competence (Ng et al., 2012; Van den Broeck et al., 2016; Vasconcellos et al., 2020). The importance of relatedness, or of one need over others, may be a question of context. While achievement contexts (i.e., school, work) may make our fundamental connection to others secondary to our own experience of competence and performance, perhaps this is not the case in other contexts such as leisure. It is also possible that the cultural context moderates the importance of relatedness, as results have shown belongingness to school being particularly important for students of color (Gray et al., 2018).

### The Role of Autonomy Support

Even though the need for autonomy was not the strongest driving factor in predicting self-determined academic motivation, teachers and parents who created an environment that supported students’ autonomy still contributed positively to the experience of self-determined academic motivation. Teacher autonomy support generally showed small but significant predictions of students’ amotivation, introjected regulation, identified regulation, and intrinsic motivation. These effects were largely mediated through the satisfaction of basic psychological needs. Still, significant small direct effects to identified regulation and amotivation were also recorded, suggesting that teachers’ autonomy-supportive behaviors may contribute to the development of those motives through other pathways. When teachers explain why a topic is important for students, it is possible that the latter will value this topic better (i.e., identified regulation) before they start feeling competent or autonomous in this class, which would explain the direct association between teacher autonomy support and identified regulation. When considering that identified regulation is a primary predictor of persistence-based outcomes (Howard et al., 2021), the current results indicate that teachers play a particularly important role in promoting persistence in educational contexts through facilitating perceived meaningfulness of educational tasks. Teacher autonomy support also directly predicts amotivation, meaning that a lack of autonomy support could be demotivating even for students who feel competent and autonomous in a subject.

In contrast with the important role of teacher autonomy support, parental autonomy support showed minor associations with student motivation. Although a moderate predictor of needs, parental autonomy support was only a weak predictor of identified and intrinsic motivation. Moreover, the association between parental autonomy support and academic motivation types were almost entirely mediated by need satisfaction. These results show that autonomy support has a largely different effect on student motivation depending on its source, which could be explained by the differing roles teachers and parents play when being autonomy-supportive. Indeed, teacher autonomy support is enacted to directly improve students’ academic experience and learning, while parental autonomy support has a more general goal of helping children grow as volitional individuals and is generally not specifically focused on their academic experience. This holistic parental influence may determine the degree of need satisfaction students begin their schooling with but ultimately loses part of its influence as students age, as shown by significant moderation effects in the current study. These expectations are in line with Vallerand’s (1997) hierarchical model of motivation that would predict school factors to be more predictive of school motivation and outcomes compared to factors occurring outside of this environment.

Taken broadly, these results validate SDT’s claim that need satisfaction in school can be a strong global predictor of student motivation. Therefore, any agent that can contribute to students’ need satisfaction at school are possibly good targets for fostering motivation in students. However, the motivational role of teachers goes beyond merely satisfying students’ psychological needs at school as indicated by the significant direct effects from teacher autonomy support to motivation types. These results concur with Hattie’s (2009) meta-analysis of 800+ student achievement predictors showing that teachers are at the forefront of learning experiences for students and are likely to have the strongest influence on student motivation.

### Introjected and External Regulation

The current results show minor associations between psychological need satisfaction and introjection. Furthermore, despite meta-analytic correlations showing a small association between both autonomy support sources and introjected regulation, these associations did not hold when needs and autonomy support predicted introjection within a single model. Since introjected regulation is associated with both adaptive and maladaptive educational outcomes (Howard et al., 2021), our results imply that need satisfaction may also carry some “hidden” negative educational consequences when it is associated with introjected regulation, although this effect is most likely very minor.

However, autonomy support and needs did not predict external regulation. Because being self-determined means not only having high levels of autonomous motivation, but also low levels of controlled motivation, the fact that the main antecedents identified by the theory do not relate to a central type of motivation begs the question: What situations influence students to become more or less externally regulated? A first possibility is that need satisfaction and autonomy support scales are not measuring the full extent of the construct. In other words, need frustration and autonomy controlling teacher/parent behaviors may be important aspects in predicting external, and possibly introjected, regulations. Need frustration variables (i.e., autonomy frustration, competence frustration and relatedness frustration) are increasingly being seen as important in SDT (Chen et al., 2015; Vansteenkiste et al., 2020). Recent results in the field of physical education indicate that they are distinct from need satisfaction and contribute to the prediction of the controlled forms of motivation (Warburton et al., 2020). However, controlling behaviors from parents and teachers have not been identified as reliable predictors of students’ external regulation (Noels et al., 1999).

### Moderating Influences

Associations between motivational types and psychological needs varied depending on participants’ characteristics. Older students seem to benefit less from parental autonomy support, showing lower associations with intrinsic and identified motivation types. This indicates that parental support might bear its highest importance as students navigate the early stages of their educational career. However, a more negative association between parental autonomy support and amotivation as students get older indicates that the importance of parental support in this regard increases over time. Amotivation is also more strongly negatively predicted by autonomy and competence as students grow older. This finding might be particularly important considering the general decline in school motivation that occurs as students progress from one grade to the next (Scherrer & Preckel, 2019). With regard to gender, samples with more male participants showed stronger associations between competence and autonomous motivation types (intrinsic and identified). This may indicate a stronger need for boys, compared to girls, to satisfy their competence in order to develop and cultivate autonomous motivation types.

Furthermore, the context, whether classroom-based or in physical education, seems to be an important moderating factor for associations between some psychological needs and motivation types. While competence in general was similarly linked to motivation types in both contexts, this was not the case for autonomy and relatedness. Their respective negative prediction of amotivation was much stronger in classrooms than in physical education. In classroom-based education, high levels of autonomy, relatedness, and competence seem important to avoid amotivation. This might not be the case in physical education where competence could be the most important factor for motivation quality. This interpretation is corroborated by Vasconcellos et al.’s (2020) meta-analytic results showing competence to outweigh autonomy and relatedness in predicting most motivation types in physical education.

Another contextual moderation was that associations between psychological needs/autonomy support and autonomous motivation types (intrinsic, identified) were much stronger in physical rather than classroom-based education. However, a similar distinction was also noted depending on scale used, where studies using the AMS had weaker associations between needs/autonomy support and autonomous motivation types compared to those using the BREQ (and PLOC). It is possible that this distinction between the two contexts is a methodological artefact derived from varying scale properties. In the same vein, the AMS shows eerily positive associations between external regulation and antecedents, an abnormal phenomenon already stressed in previous research (see Howard et al., 2017, Figure 5).

### Practical Implications

Results of this meta-analysis reify the importance of teacher autonomy support for students’ autonomous motivation. Specifically, they show that regardless of age, school level, nationality, or gender, autonomy support predicts autonomous types of motivation, thereby providing support for existing interventions designed to increase student need satisfaction and motivation through autonomy-supportive practices (Su & Reeve, 2011; Vasquez et al., 2016). In line with our results, these interventions will be especially effective if they target teachers in the first place instead of parents. However, solely focusing on more training is not enough. There is a need for a more careful attention on the features of schooling that constrain the autonomy support that teachers can give to students. It is well-known that teachers face various pressures from employers and students that may limit their capacity to support student’s autonomy (Pelletier et al., 2002). For example, high-stakes testing or pay-by-performance for teachers may put pressure on teachers that could limit their capacity to provide autonomy support: They may be more tempted to use controlling strategies such as rewards and punishment to try and coerce students into attaining various imposed educational goals. Thus, even if professional development programs have demonstrated their usefulness in shaping teacher autonomy-supportive practices (Guay et al., 2020), we argue that further improvements are needed to create the conditions in which teachers possess the effective tool to bring change to their classroom. Implementing these programs in a context of high pressure is likely to be a waste of resources given the structural inability for teachers to implement the acquired qualifications. Rather, reforms from bottom-up helping teachers adapt their practice (Darling-Hammond, 1994) would allow the necessary margin needed to integrate autonomy-supportive teaching practices.

Our results also emphasize the importance of the need for competence. In this regard, teachers may need to go beyond autonomy-support to embrace specific behaviors known to increase student competence, such as high-quality feedback (Hattie & Timperley, 2007). According to Hattie and Timperley (2007), the main purpose of feedback is to diminish the gap between students’ current performance and their expected performance. Whether it helps students develop their skills or reach more realistic expectations, both situations are expected to increase the need for competence (Ryan & Deci, 2017). While this suggestion is speculative because our findings do not focus on teacher feedback, it is well anchored in the field of motivation (Wisniewski et al., 2020).

### Limitations and Conclusion

A major limitation of this meta-analysis is that it is based on correlations, limiting conclusions regarding causality. Specifically, even though each part of the tested model (Figure 2) has previously been tested and some causal links verified (Froiland, 2011; Guay et al., 2016; Hausmann et al., 2009; Patall et al., 2010), the current study cannot speak definitively on the issue of causality as our results may be attributed to other variables. Future research should thus move away from cross-sectional data and employ more sophisticated methodological designs including longitudinal, experience sampling, and quasi-experimental designs.

It must be noted that our search found relatively few studies examining the association between parental autonomy support and academic motivation types. Likewise, autonomy support from peers was studied in very few articles falling within our literature review and was therefore not included in main analyses. This shortcoming represents a gap in the literature. Additionally, the limited body of research addressing teacher and parental control, as well as other need-supportive behaviors (competence support and relatedness support; Ratelle et al., 2021) restricted our ability to implement these variables in the current analysis, even though they fall within the theoretical scope of motivational antecedents theorized by SDT. This was also the case for need frustration variables (Vansteenkiste & Ryan, 2013). A further limitation concerns possible publication bias. Even though steps were taken to collect unpublished work, and estimates correcting for publication bias indicate only minor influences, the fact remains that estimates relating to teacher autonomy support were possibly inflated in the current study due to this bias. As such, results concerning this variable should be interpreted with caution. Further meta-analytic research building on this study in the future may be helpful in clarifying this issue.

In conclusion, the present investigation comprehensively reviewed the theoretical antecedents of student motivation through the perspective of SDT. As expected, results highlight that autonomy-supportive behaviors from teachers and parents, as well as autonomy, competence, and relatedness satisfaction, are all generally associated with students’ self-determined motivation. More specifically, satisfaction of the competence need was found to be particularly highly related with intrinsic and identified motives, indicating its centrality in the education context. Teachers’ autonomy support was the strongest predictor of motivation through need satisfaction, although parental autonomy support was a positive but weaker antecedent, ultimately implying the importance of proximal sources of support.

## Notes

The full dataset and all associated supplementary materials are made available through Open Science Framework. All files can be found with the following link: https://osf.io/pje9t/?view_only=b8028e21a5f3450383e368f3668b9245 This study was made possible through funding from the Social Sciences and Humanities Research Council of Canada to the first and fourth authors and from the Fonds de recherche du Québec–Société et culture to the first author, and internal funding from Monash Business School awarded to the second author. We thank Geneviève Morneau-Vaillancourt for her help in gathering part of these data.

1 Other candidate antecedents such as need frustration as well as competence/relatedness support were also included in the literature search. However, their low rate of inclusion in studies measuring student motivation prevented us from including them in the analysis as antecedents.

2 In the Lv and Maeda (2020) appendix showing full code for this procedure, there is a typo that short-circuits the multiple imputation. This was corrected in the present analysis. See supplemental material for the full R code for this analysis.
